# Place-based approaches to improve the mental health and wellbeing of children and young people: a rapid realist review

**DOI:** 10.1186/s13643-025-02838-8

**Published:** 2025-05-19

**Authors:** Anna March, Kate Allen, Karuna Davies, Julie Harris, Alison Bethel, Laura Kennedy, Tamanna Malhotra, Rachael Stemp, Bianca Alexandrescu, Tim Hobbs, Peter Fonagy, Steve Pilling, Vashti Berry

**Affiliations:** 1 Children and Young People’s Mental Health Research Collaboration, University of Exeter, Exeter, UK; 2 Department of Clinical, Educational, and Health Psychology, UCL, London, UK; 3 Dartington Service Design Lab, Buckfastleigh, England UK; 4 Anna Freud, London, UK

**Keywords:** Systemic change, Social determinants, Mental health, Wellbeing, Realist review

## Abstract

**Background:**

Adolescent mental health continues to be a pressing public health concern across the globe, in spite of renewed efforts in recent years to improve mental health and wellbeing outcomes. With many mental health services and systems ill-equipped to meet young people’s needs, there is growing evidence that prevention interventions addressing the social and structural determinants of mental health should be prioritised. Consequently, there is a move away from isolated interventions towards ‘place-based approaches’ that aim to unlock systemic or structural change. However, there is limited evidence on how these approaches create change and the different contextual factors or underlying mechanisms that influence outcomes. This is the first realist review on place-based approaches to improve young people’s mental health.

**Methods:**

This rapid realist review synthesises relevant literature on place-based approaches to improve the mental health and wellbeing of children and young people. The review involved consultation with programme developers, an expert panel of content specialists and a Young Person’s Advisory Group. Online databases were searched for peer-reviewed and grey literature published between January 2000 and August 2023, resulting in 5685 articles.

**Results:**

Fifteen articles from eight countries were included in the review, from which 11 realist context-mechanism-outcome configurations (CMOCs) were developed to explain the underlying mechanisms present in the place-based approaches. These CMOCs were categorised into three themes: (1) Building relationships and trust, (2) bringing a social determinants lens, and (3) educating and empowering community stakeholders.

**Conclusions:**

This review provides valuable insights into the mechanisms underpinning place-based approaches. However, the mechanisms identified primarily address the intermediate outcomes of place-based approaches, such as engaging the right stakeholders and creating opportunities for youth voices to be heard. Articles did not address the challenges around long-term sustainability and there remain crucial questions around the pathway to wider systemic change. Recommendations are included for future development and evaluation of place-based approaches to improve young people’s mental health.

**Systematic review registration:**

PROSPERO CRD42023450778.

**Supplementary Information:**

The online version contains supplementary material available at 10.1186/s13643-025-02838-8.

## Background

Despite an increased focus in many countries on improving mental health and wellbeing outcomes for young people, adolescent mental health continues to be a key public health issue across the globe [41, 56]. In England, recent studies indicate that up to 20% of young people may be experiencing mental health issues [42]. The ramifications of early mental health problems are extensively documented and include adverse effects on physical health, educational success, substance use, criminal behaviour, employment prospects, not in education, employment or training (NEET) status, and economic challenges [4, 20, 45, 54]. Consequently, the global community has recognised the urgency of investing in child and adolescent mental health and wellbeing, highlighting the need for cross-sector approaches [33, 56].

However, many mental health services and systems are not adequately prepared to address the needs of the population, with governmental funding frequently allocated to other health areas and mental health budgets mainly supporting inpatient care [56]. Nevertheless, there is accumulating evidence that efforts in promotion and prevention can be cost-effective and that strategies targeting the social and structural determinants of mental health ought to be given priority [1, 6, 36, 56]. Recognising the complexity and the impact of social determinants on the mental health of children and adolescents, certain programmes are transitioning from isolated interventions to methods that seek systemic or structural shifts. Typically known as “place-based approaches”, these initiatives are usually rooted in specific geographies and emphasise sustained collaboration and partnerships [2, 48]. Place-based approaches can take a number of different forms, from government-led regeneration in a specific geographical area, to local partnerships that focus on issue-based change [48]. While the collaborators will vary according to the specific goals of a place-based approach, these initiatives involve long-term efforts across multiple organisations or actors to create structural change. There is often a focus on building and developing relationships in a community and this work is characterised by partnerships and shared design, as well as shared accountability for impact [11]. There is limited research, however, into how these approaches effect change and the various contextual elements or underlying mechanisms that influence the outcomes of this work.

This rapid realist review aims to consolidate current evidence on place-based approaches for enhancing the mental health and wellbeing of young people. It is an integral component of a broader realist evaluation of the Kailo programme, a research and design effort focused on developing and deploying context-specific preventive measures to improve adolescent mental health in parts of the UK [24]. The theory-driven realist methodology is particularly suited to complex and large-scale programmes as it embraces complexity and facilitates the examination of interwoven contextual factors, causal mechanisms, and outcomes [50]. Moreover, the rapid realist review method offers a practical output for researchers and policymakers when faced with constrained time and resources [49].

The insights and suggestions derived from this rapid review have been used to inform the development of the initial programme theories which explain how and why the Kailo programme works, under what conditions, and for whom. For full details of the Kailo programme see Hobbs et al. [24]. While the findings are of direct value to inform this programme’s development, they also provide valuable insights for others looking to develop and deliver place-based systemic change in this field.

## Methods

The review protocol was registered on PROSPERO in August 2023 (ref: CRD42023450778). We conducted our review in keeping with Saul et al.’s [49] guidance on rapid realist review methodology, which modifies Pawson’s [46] realist synthesis for expedited analysis to ensure a time-sensitive process. Realist reviews are a theory-driven approach to synthesising research that answer the questions ‘what worked, for whom and in what circumstances, how and why?’. To direct our reporting, we followed the RAMESES publication standards for realist syntheses [55], detailed in Additional file 1. Table 1 provides brief definitions of key realist concepts (in line with Jagosh et al. [27]).

**Table 1:** Definition of terms

Realist term	Definition
Context-mechanism-outcome configuration (CMOC)	Explanatory statements for outcomes in the observed data. The process of creating these configurations draws out the relationship of context, mechanism and outcome of interest in a particular programme. A CMOC can reference either the whole programme or only certain aspects.
Context	The “backdrop” of programmes and research. Examples include geographic location (e.g. urban or rural), cultural norms and history of a community, existing social networks, funding or infrastructure. Contexts also operate in time, and programme activities may change the context while the programme is being implemented.
Mechanism	The interaction between programme resources and the ways that participants interpret and respond (or do not respond) to them. This is the generative force that leads to outcomes. For example, in this review, mechanisms may refer to why young people or community professionals choose (or choose not) to participate in a place-based approach.
Outcome	Either intended or unintended and may be intermediate as well as final. Outcomes are broader than the specific goals of a given programme. Over the course of programme delivery, outcomes can become the context for new activities or mechanisms. This is often described as a ‘ripple effect’ [27].

Consistent with Saul et al. [49], the review involved consultation with stakeholder groups—including the broader Kailo programme team and a Young Person’s Advisory Group—as well as a panel of external content specialists (see Table 2 for membership of these groups). This collaborative effort aimed to enhance the review's applicability and relevance to contemporary practices

**Table 2:** Stakeholder groups

Group	Members
Wider Kailo team	This includes members of the Kailo consortium outside of the evaluation team (who led on this review). Namely those who have been directly involved in the design and delivery of Kailo or who have responsibility for governance and strategic direction.
Young Person’s Advisory Group	Thirteen individuals aged 16–25 from Northern Devon and Newham, London (the two sites of the first phase of Kailo work).
Expert panel	Four academics with expertise in either place-based approaches and/or realist reviews in the field of public health.

### Searching processes

Relevant literature and documents were sourced through searches using specific bibliographic databases, citation searching, and consultation with our expert panel and the Kailo team. The database search was conducted in two phases.

In the first phase, a preliminary scoping search was based on four papers [12, 29, 44, 51] identified through conversations with key stakeholders and some initial searches using Google Scholar. We performed forward and backward citation searching on these articles on Web of Science and Google Scholar, followed by an extensive search in the Cochrane Library (via Wiley), Epistemonikos, Social Science Citation Index (via Web of Science), and PsycINFO (via Ovid), focusing on place-based initiatives targeting the mental health of children and young people on 1 st August 2023. The search strategy for this phase was developed with inputs from the review team, the wider Kailo programme team, our expert panel, and an information specialist (AB). This scoping search combined terms relating to place-based approaches, children and young people, and mental health and wellbeing. AM, KA, and TM reverse-screened the first 100 results from this search (beginning with the latest publications) to identify potentially relevant studies, which were then reviewed in detail with the entire review team. This helped with the development of the second phase of the search and the inclusion and exclusion criteria.

The second phase involved a more detailed search strategy, formulated with the review team and the expert panel. The search terms were refined to encompass broader aspects of place-based strategies and included keywords for ‘participatory approaches’ (detailed in Additional file 2), a concept identified as closely related to place-based strategies in the initial search phase. Searches were carried out in PsycINFO (via Ovid), Social Science Citation Index, Social Sciences and Humanities Conference Proceedings, and Emerging Sources Citation Index (via Web of Science) in August 2023. This comprehensive search aimed to include both academic and grey literature. We limited our search to documents published in English from 2000 onwards, with a view to capturing the most recent and relevant literature on place-based approaches to support adolescent mental health and wellbeing [39].

### Selection and appraisal of documents

Search results from the second stage were downloaded into Endnote, de-duplicated and uploaded to Rayyan [43] for the processes of screening, selection, and appraisal. The screening of titles and abstracts was carried out by AM, KA, and KD, guided by the inclusion and exclusion criteria specified in Table 3. Each title and abstract was screened by a single researcher, with an random 10% of these independently reviewed by a second reviewer (TM or LK). The review team met regularly to discuss the title/abstract screening and addressed any discrepancies through collective discussion.

**Table 3:** Screening inclusion/exclusion criteria

	Inclusion criteria	Exclusion criteria
Population	Children and young people (aged ≤ 25 years old).	Adults (aged > 25 years old) or interventions not specifically for children and young people (e.g. universal interventions targeting all age groups).
Intervention	A place-based approach, programme, intervention, or set of activities. The place-based approach must be collaborative (involving more than one organisation) and focus on creating structural or systems change.	Any approach, programme, intervention, or set of activities that involves only one organisation or does not focus on creating structural or systems change.
Main aim(s)	Main aim(s) must include improving mental health and/or wellbeing (defined as common mental health problems, severe mental health problems, substance misuse disorders, and constructs of emotional wellbeing).	Any approach, programme, intervention, or set of activities that does not aim to improve mental health and/or wellbeing as defined opposite.
Study designs	Any study design that is relevant to our research questions (e.g. quantitative, qualitative, implementation and intervention studies).	N/A
Language	English.	Any record not published in English.
Dates	Records published between 2000 and 2023.	Any record published before 2000.

Full-text screening was conducted by AM, KA, and KD, first assessing each article against the inclusion/exclusion criteria (Table 3). Again, each text was screened by a single author, with a random 10% screened in duplicate and any discrepancies discussed and resolved amongst the team. While there are varying definitions of ‘young people’, ‘youth’ and ‘adolescence’ in the literature, we adopted the same maximum age range as the Kailo programme (up to and including 24 year olds) in order to identify relevant articles.

This first screening process was followed by a deeper consideration of the relevance, richness and rigour of the article [9, 26, 55]. Realist synthesis aims to build theories by searching diverse data sources for insight into the nature of different initiatives [26, 47]. An article’s relevance was determined by its contribution to the development and testing of context-mechanism-outcome configurations (CMOCs). Questions to assess relevance included: Does the article describe the programme’s architecture in sufficient detail? Does the article contain at least one statement regarding how, for whom, why the programme works? Richness was then scored in relation to some of the key themes underpinning place-based approaches, as identified by the wider Kailo programme team in their description of the Kailo framework [24]. These overarching themes included trust, effective collaboration, ownership, capacity, evidence integration, and co-design. Rigour was examined by considering the trustworthiness of the data, i.e. the coherence between data collection methods and the generated data [15]. Articles assessed as having low relevance and/or methodological rigour were not necessarily excluded from the review, but those with high relevance and richness were prioritised for data extraction, with the lower scoring articles referred to later in the process to further test and refine the CMOCs.

### Data extraction, analysis and synthesis

An initial data extraction template was created in Excel to document geographical location, the characteristics of the place-based approach (including its objectives, target population, and structure), and its underpinning theories or values. The articles were then imported into NVivo [35] and coded by either KA or AM into preliminary CMOCs, guided by existing recommendations on using NVivo in realist evaluations [10, 19]. This was an iterative process, characteristic of all realist reviews [46, 49]. We started with the most relevant and rich papers and added others as the CMOCs were developed. We also used backwards and forwards citation and website searches to find further reports and studies.

Throughout the data extraction and analysis process there was an ongoing dialogue with the Kailo stakeholder group and our expert panel. Once more concrete CMOCs were developed, these were also shared with our Young Person Advisory Group (YPAG). For the young people to engage meaningfully in the review process, we first held two sessions to provide training on realist research methods and the aims of the Kailo programme. The YPAG then met online and were shown early versions of the CMOC wording, along with potential diagrams and explanations of the ripple effects. The authors also held online and in person meetings with the expert panel to discuss the framing of the results and to explore certain elements of CMOCs in more detail. Feedback from all groups was instrumental in refining the CMOCs, offering insights into mechanisms and proposing additional contexts or outcomes for further investigation in the literature. For example, CMOC 9 was initially based around a mechanism of young people feeling more valued as a result of taking part in these place-based approaches. However, members of the YPAG emphasised the importance of young people being able to build confidence and communication skills. Further exploration in the literature suggested that this was indeed the key mechanism for young people to feel empowered to take further action.

## Results

**Fig. 1:**
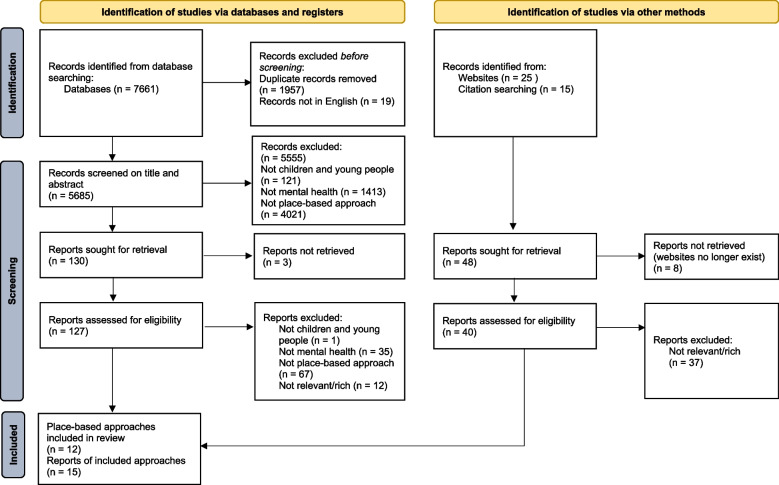
PRISMA diagram

The PRISMA diagram in Fig. 1 shows the flow of information through the rapid review process. In total, 15 articles on 12 distinct programmes or place-based approaches were included for data extraction. Table 4 provides a descriptive overview of the included studies.

**Table 4:** Descriptive overview of included studies

Intervention						Articles and documents reviewed (source)
Name	Country	Aim	Participants/people	Underpinning theories	Activities/processes	
1. Agenda Gap	Canada	Equip youth for meaningful policy engagement focused on the social determinants of health and equity, to strengthen mental health and wellbeing for individuals and their communities	- Youth collaborators (15–24 years) in British Columbia and Alberta (emphasis on engaging youth who experience intersecting health and social inequities) - Adult allies and mentors from community organisations, non-profits, health authorities, school boards - Service providers and policymakers	- Mental health promotion - Positive youth development - Community youth development - Theory of Sociopolitical Development (liberation psychology) - Trauma and violence informed practice - Social determinants of health	1. Youth mental health promotion and policy advocacy “curriculum” delivered through a developmental relationship building process 2. Facilitator and ally capacity-generating activities (community, policy and other decision maker) 3. Strategic knowledge mobilisation	Jenkins et al. [29] *Advancing socioecological mental health promotion intervention: A mixed methods exploration of Phase 1 Agenda Gap findings* (citation searching)
2. Barnardo’s strategic partnership work	UK	Support system change/transform systems in child and adolescent mental health, encourage innovation/new ways of working, promote the mental health and wellbeing of children and young people, and reduce service demand by increasing preventive efforts	Barnardo’s strategic partnerships including North Tyneside, Renfrewshire, and South-Eastern Trust Each partnership involved: - Children, young people, and families - Health, education, local government and third sector organisations	- Systems thinking and theory of change approaches	Each partnership: 1. Mapped current service provision and need with local stakeholders (see participants) 2. Co-produced ways to promote transformational change	Doherty et al. [12] *Developing a theory of change methodology to support the evaluation of place-based systems change interventions to support child and adolescent mental health and well-being* (database searching)
3. Building Bridges	Australia	Develop a co-designed work-practice model for mental health care on Whadjuk Nyoongar country in Perth, Australia	- Elders and Aboriginal young people (between 16 and 25 years) - Service partners (30 non-Aboriginal staff members, five Aboriginal staff members) - Policy and advocacy organisations (seven non-Aboriginal, one Aboriginal)	- Indigenous research methodologies - Participatory Action Research	1. Hiring co-researchers 2. Cultural engagement activities (*Storying* activity, *On Country* event) 3. Group yarns 4. Prioritisation exercise 5. Designing the model together	Culbong et al. [8] *Building a Nyoongar work practice model for Aboriginal youth mental health: Prioritising trust, culture and spirit, and new ways of working* (database searching) Wright et al. [57] “*If you don't speak from the heart, the young mob aren't going to listen at all”: An invitation for youth mental health services to engage in new ways of working* (citation searching)
4. HeadStart	England (UK)	Explore and test new ways to improve the mental health and wellbeing of young people aged 10–16 and prevent serious mental health issues from developing	- Local-authority-led HeadStart partnerships in Blackpool, Cornwall, Hull, Kent, Newham and Wolverhampton - Young people (10–16-year-olds), schools, community providers (youth services, VCS, social care, health, CAMHS)	- Ecological framework - Systems thinking	Each partnership developed, commissioned, delivered and evaluated portfolios of support for young people, co-designed with young people	Stapley et al. (2023) *Early adolescents'experiences of a school- and community-based prevention program: perceived'bridge's and'walls'to promoting mental health and wellbeing* (database searching) Stapley and Burrell [52] *Changes in local areas as a result of the HeadStart programme: stakeholder perspectives* (grey literature) Evidence Based Practice Unit [16] *Heads Up: How are systems change and sustainability being approached in HeadStart?* (grey literature)
5. Jigsaw	Ireland	Engage communities in systematic and data-based processes of planning, programme design, implementation and evaluation to improve youth mental health services and supports in the Republic of Ireland	- Five demonstration sites across four of Ireland's counties - Community coalitions included young people (12–24), family members, community leaders, educators, youth workers, specialised mental health workers, local health manager responsible for statutory services	-Developmental-ecological framework (Bronfenbrenner, 1977) - Literature on: - Comprehensive community initiatives - Systems of care - Integrated services - Developmental asset building - Youth engagement and participation - Positive youth development	1. Assess community readiness and creating demonstration sites (developing business plan, assembling financial and human resources required for implementation) 2. Comprehensive assessment of needs and resources (focus groups, key informant interviews, community and provider surveys, reviewing local service data, geo-mapping of resources) 3. Delineate needs of young people 4. Determine comprehensive community goals 5. Consider evidence-based intervention alternatives 6. Create logic model and theory of change 7. Delineate implementation and evaluation plans 8. Monitor and revise initiative	Illback et al. [25] *Jigsaw: Engaging communities in the development and implementation of youth mental health services and supports in the Republic of Ireland* (database searching)
6*. Mamow Sha-way-gi-kay-win*	Canada	Model meaningful relationships between non-Aboriginal and First Nations’ people to influence public funders, philanthropy, governmental and non-governmental sectors as they negotiate relationships and resource development; to subsequently enhance the social determinants of health and overall lives of Aboriginal people in Northern Ontario	- First Nation chiefs, Elders, youth and community members in Northern Ontario - 100 individuals and voluntary organisations in Southern Ontario	- Social determinants of health	1. Community needs assessments (needs, strengths, challenges, existing resources, history and story of communities, as told by the communities themselves) led by experts in mental health, child and youth care, justice, economic development and housing 2. Research consortium of 15 academics to guide the use of the social determinants of health framework and to examine existing literature and relevant models	Finlay et al. [17] *Mamow Ki-ken-da-ma-win: A partnership approach to child, youth, family and community wellbeing* (database searching)
7. *Nges Siy* (I love you)	Canada	Build capacity of nations to define the issue of suicide through their lens and to develop community-specific interventions to address youth suicide	- Elder and youth representatives (15–24 years old) from 11 communities in Northern British Columbia - Carrier Sekani Family Services (health, social, legal and research services) - University of Northern British Columbia	- Indigenous research methodologies - Participatory research	1. Community consultation meeting 2. Cultural camps for young people 3. Development of a suicide awareness and prevention manual and accompanying training	Harder et al. [21] *Nges Siy (I love you): A Community- Based Youth Suicide Intervention in Northern British Columbia* (database searching)
8. Right care, first time, where you live	Australia	Engage local communities using systems science methods to mobilise knowledge and action to strengthen youth mental health services	Range of community stakeholders including those with lived experience, carers, and Aboriginal and Torres Strait Islander people, and youth mental health system decision-makers	- Participatory Action Research - Shared decision-making processes - Knowledge mobilisation - Participatory system modelling - Community engagement framework	1. Site selection and capacity to participate 2. Establishing relationships and building trust 3. Site visits (a) leadership stakeholders to discuss mental health needs, networks and political landscape, (b) operational team to discuss mental health challenges in local context and participatory modelling process, (c) technical team to discuss systems modelling and identify local stakeholders who will be trained 4. Committing to meaningful engagement (researchers) 5. Committing to the process (stakeholders)	Freebairn et al. [18] *Applying systems approaches to stakeholder and community engagement and knowledge mobilisation in youth mental health system modelling* (database searching)
9. Seven Circles Coalition	Alaska, USA	Develop, implement and evaluate a youth/adult partnership model for a regional substance abuse prevention coalition	- Representatives from community-based substance abuse prevention organisations in three Southeast Alaska communities - Community youth representatives (and youth co-chair of steering committee)	Youth/adult partnership model	1. Setting up community partnerships to develop prevention approaches in their communities (e.g. local radio programmes, teen recreation and activity centre) 2. Networking, training and technical assistance for each community partnership (including sustainability and evaluation training)	Wunrow et al. [58] *Promoting youth/adult partnerships: The seven circles coalition in Sitka, Alaska* (database searching)
10. Strategic Prevention Framework	USA	For young people to collectively engage in a planning process to create and implement a strategic plan that uses evidence-based strategies to create community-level change	- Young people (12–17-year olds) in high schools - Adult allies - Ohio Department of Mental Health and Addiction Services (funders of the work)	- Youth Empowerment Conceptual Framework - Substance Abuse and Mental Health Services Administration's Strategic Prevention Framework	1. Youth Council 2. Training adult allies to lead and support young people (and to facilitate/convene the groups) 3. Learning community meetings and ongoing training and technical assistance for adult allies	Collura et al. [5] *Creating spaces for young people to collaborate to create community change: Ohio's youth-led initiative* (database searching)
11. Teenage Mothers Project	Uganda	Community-based empowerment intervention to improve psychological and social well-being of unmarried teenage mothers in rural Uganda	Charity volunteers, health promotion academics, community leaders and unmarried teenage mothers (15–19-year-olds)	- Intervention Mapping - Participatory intervention planning - Ecological approaches	1. Needs assessment 2. Creating a matrix of change objectives 3. Selection of methods and programme design 4. Planning for adoption, implementation and sustainability (identifying outcomes and performance objectives for stakeholders on all levels) 5. Planning an evaluation	Leerlooijer et al. [34] *Applying Intervention Mapping to develop a community-based intervention aimed at improved psychological and social well-being of unmarried teenage mothers in Uganda* (database searching)
12. Youngballymun	Ireland	To embed EBPs, on an area-wide basis, within usual care services for disadvantaged children and young people living in Ballymun, Dublin	Local service providers, service executives and managers, local residents, children and young people (up to 20 years old), parents	- Implementation theories (e.g. diffusion of innovations theory, CFIR) - Powell (2015)–Implementation strategies	1. Capturing the ‘community voice’ 2. Capturing ‘children’s voices’ 3. Auditing existing services within the community 4. Reviewing existing evidence of what works (i.e. identifying evidence-based services) 5. Conducting a local needs analysis 6. Reviewing evidence of existing need 7. Reviewing the national policy context 8. Engaging with key stakeholders and decision makers	Hickey et al. [23] *Strengthening stakeholder buy-in and engagement for successful exploration and installation: A case study of the development of an area-wide, evidence-based prevention and early intervention strategy* (database searching)

**Fig. 2:**
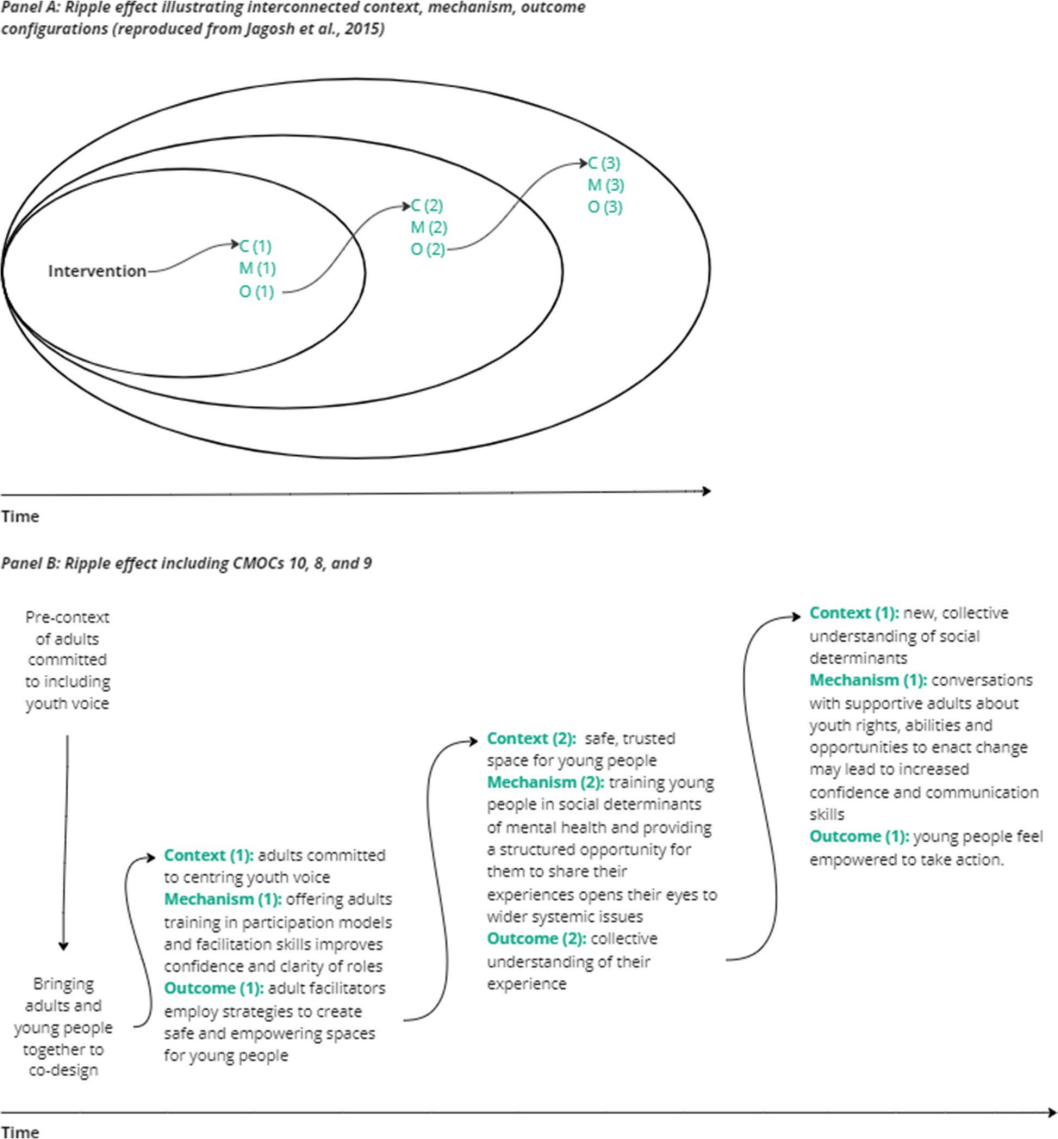
Ripple effects

Table 5 is a list of the 11 CMOCs developed from the included articles. After discussions with stakeholders and our panel of experts, it was agreed that although many CMOCs are interconnected, they can be categorised into three main themes: (1) building relationships and trust, (2) bringing a social determinants lens, and (3) educating and empowering community stakeholders. Each CMOC is explored further in the following sections, supported by relevant findings from the literature. Figure 2 illustrates ripple effects within the 11 CMOCs identified, whereby the programme activities can be viewed as catalysts within the system, ‘leading to the evolution of new structures of interaction and new shared meanings’ [22], p. 267). The outcomes of some of the CMOCs become the context for new activities or mechanisms over time.

**Table 5:** List of CMOCs

Category	Number		CMOC	Key recommendations for place-based approaches
Building relationships and trust	1		When adults are aware of the importance of creating validating and safe spaces (C), facilitators thinking carefully about language and inclusion can make the space feel non-judgemental and accessible (M), helping young people to share their experiences openly and honestly (O).	- Adults need to select language carefully to promote inclusivity and respect (avoid complex terminology) - Adults should take time to hear from young people about what they need for a space to feel safe and accessible, and tailor accordingly
	2		Where there are cultural differences between the programme team and local communities (C), the programme team engaging in a community’s way of knowing and demonstrating reflexivity can lead to a deeper understanding of everyone’s worldview (M). This can help to establish trust and meaningful connection between the team and communities (O1) and may begin to address power imbalances (O2).	- Programme teams should demonstrate reflexivity - Programme teams should show a commitment to embracing diverse methodologies in their work
	3		Where new programmes are launched (C), the programme team visibly involving trusted and recognised members of the community in programme activities can lead to stakeholders perceiving the programme as valuable (M). This perception can lead to greater engagement from community professionals and young people in the work (O).	- Programme teams should work with influential individuals within a community (e.g. respected Elders, or frontline youth workers) to establish credibility and authenticity
	4		Where new, long-term programmes are introduced (C), having sufficient time for the initial stages creates space for the programme team to comprehensively understand the local ecosystem (M). This understanding can facilitate the building of trusting relationships between the programme team and local community members (O).	- Programme teams, especially external academic researchers, must allocate enough time in the early phases to cultivate trusting relationships with community members
	5		Where programmes have allocated ample time (C1) and programme teams are open to learning and adapting (C2), adopting a flexible strategy in planning and programme development provides the opportunity for community voices to be heard (M). This adaptability ensures the initiative is more attuned to community needs (O1) and facilitates the integration of change within the community (O2).	- Programme teams should take a flexible and evolving approach to their activities, demonstrating an openness to change plans in response to the community
	6		Where multiple different organisations come together for the first time (C), programme teams facilitating the development of a shared vision can help create a collective understanding of local issues and the focus of the programme (M) and may lead to a stronger foundation for partnership working (O).	- It is important to create a shared vision or mission for the specific work of the place-based approach, and to communicate this clearly and consistently over time
Bringing a social determinants lens	7		In contexts where the prevailing focus is on an individualistic model of mental health (C), introducing programmes with a deliberate emphasis on social determinants facilitates a broader understanding that encompasses environmental factors (M). This approach can help community professionals think more holistically about young people’s mental health (O).	- Programme teams should frame youth mental health as affected by multiple factors, histories and systemic influences and explain how the work is distinct from conventional service delivery
	8		In the context of a safe, trusted space (C), training young people in social determinants of mental health and providing a structured opportunity for them to share their experiences opens their eyes to wider systemic issues (M) and helps to create a collective understanding of their experience (O).	- Programmes should educate young people on the social and structural determinants of mental health and provide opportunities for them to contextualise their personal experiences within a broader system
	9		With a new understanding of social determinants (C), conversations with supportive adults about youth rights, abilities and opportunities to enact change may lead to increased confidence and communication skills (M), helping young people feel empowered to take action (O).	- Adults should provide opportunities for learning and discussion to help build confidence, self-esteem and leadership skills in young people
Educating and empowering community stakeholders	10		Where adults are committed to centring youth voice (C), offering them training in participation models and facilitation skills improves confidence and clarity of roles (M), enabling them to employ strategies to create safe and empowering spaces for young people (O).	- Programme teams should not assume that local professionals (e.g. social workers, teachers, youth workers) will have the necessary skills to facilitate meaningful youth participation, and should provide training accordingly
	11		Where system leaders traditionally do not involve youth in strategic planning or decision-making processes (C), providing opportunities to see the process and outputs from activities with young people means they may see the value of youth voice (M) and shift their practice to include young people in mental health strategy design (O).	- Programme teams should provide opportunities for system leaders to be involved in youth voice activities and participatory research

### Building relationships and trust

The importance of building relationships and trust was a recurrent theme across all articles in the review. This ranged from the individual level (e.g. how adults build trust with young people in co-design activities) to an organisational level (e.g. the importance of creating a shared vision to improve partnership working). The articles included in the review all involved new programmes starting in an area, often led by external teams of researchers or mental health service staff who were new to the community, and emphasised the time taken to build trusting relationships with community stakeholders.

#### CMOC 1: When adults are aware of the importance of creating validating and safe spaces (C), facilitators thinking carefully about language and inclusion can make the space feel non-judgemental and accessible (M), helping young people to share their experiences openly and honestly (O)

Many of the included articles described adults and young people coming together to discuss the problems around youth mental health and wellbeing in their local area and to prioritise potential solutions. Given the inherent power imbalance between adults and young people in these interactions, authors highlighted the need to create non-judgemental and accessible spaces for young people to engage in this type of place-based work [5, 8, 18, 29]. Freebairn et al. [18] emphasise the importance of carefully selecting words and language in the programme to promote inclusivity and respect, avoiding any potential for additional disadvantage or social exclusion. They advocate for language that is suitable for all ages, respectful, non-judgmental, free of complex terminology, and understandable to individuals from any socioeconomic or educational background [18], p. 9). Similarly, the Agenda Gap project in Canada had an explicit focus on including young individuals facing multiple health and social challenges, with the researchers also dedicating time to interview all interested youths to “curate cohorts of 5–15 youth with shared experiences or interests” [29], p. 3).

“Many participants described the role of safety and nonjudgement in supporting them to be open and honest about what they were experiencing… ‘It’s the first place where I have adults or other students that I can openly talk to about my experiences or what is happening around us and not have to walk on eggshells, making sure what I said didn’t offend anyone. Because all the time in this group, I felt supported. I felt validated’ [Participant].” [29], pp. 8–9)

#### CMOC 2: Where there are cultural differences between the programme team and local communities (C), the programme team engaging in a community’s way of knowing and demonstrating reflexivity can lead to a deeper understanding of everyone’s worldview (M). This can help to establish trust and meaningful connection between the team and communities (O1) and may begin to address power imbalances (O2)

Several articles from Canada and Australia describe programmes that involve external researchers or staff involved in mental health service delivery working with Indigenous^1^ Canadian or Australian communities [8, 14, 17, 18, 21, 57, 58]. Often in these areas the research and service delivery staff include no or very few Indigenous Canadian or Australian people, leading to significant cultural gaps between the programme facilitators and the communities they aim to serve [18, 21]. In this context, the literature stresses the importance of the programme team’s reflexivity and their commitment to embracing and integrating diverse methodologies into their work, such as Indigenous Australian research approaches and epistemologies [8, 21, 57]. These practices serve as a foundation for relationship-building and establish trust and safety between the programme team and community members [8]. It is important to note that these examples draw on striking cultural differences among communities and service providers in Australia and Canada. In other contexts, such cultural differences are likely more subtle and less frequently acknowledged, requiring deeper reflection from programme teams to bring differences and power dynamics to the fore.

“The first stage of the co-design process involved group yarns within the participant group to begin the process of meaningful engagement. Yarning is recognised as both a cultural and safe way for relating and communicating on sensitive topics, using reciprocity as its primary focus; it requires the researcher to develop and build a relationship that is accountable to Aboriginal people participating in the research.” [8], p. 178)

“There was an investment in establishing safe spaces for the Elders, young people and service providers to engage in open, honest dialogue. Safe spaces in this context requires service providers having a heightened awareness of their role and actively addressing the power imbalances between young people and themselves as clinicians and service managers.” [57], p. 1508)

#### CMOC 3: Where new programmes are launched (C), the programme team visibly involving trusted and recognised members of the community in programme activities can lead to stakeholders perceiving the programme as valuable (M). This perception can lead to greater engagement from community professionals and young people in the work (O)

Although all reviewed articles underscore the critical role of community member involvement for the success of place-based initiatives, several studies emphasised the importance of engaging specific influential individuals to establish credibility and authenticity within a community [8, 18, 34, 57]. Again, this was particularly pertinent in Indigenous Canadian or Australian communities, where participation of Elders was identified as crucial [8, 18]. In the Building Bridges project based in Perth, Australia, the six Nyoongar Elders who were engaged in the design process were identified for recruitment by community members [8]. Culbong et al. [8] referred to Aboriginal Elders and young people as ‘co-researchers’ to emphasise the significance of their contributions not only to the project’s outcomes but also to its collaborative and consensus-driven processes (p. 176). Moreover, the opinions of Elders and young people were also given extra weighting in the form of additional votes in the priority setting process that informed the design of the work practice model.

“The Aboriginal Elders are recognised as the traditional custodians of culture and leaders in their community, and their status naturally affords them a position of authority. Engaging Elders provides cultural security, authenticity and legitimacy to service change… Aboriginal young people who are recognised by their communities as emerging leaders, must be at the forefront of initiatives that impact them and their families and communities in which they live.” [8], p. 182)

In contrast, Hickey et al. [23] described a situation in Dublin, Ireland, where many community professionals did not feel that the programme team had involved or consulted recognised members of the community. The authors noted substantial resistance from service providers in the area, as people felt that only senior managers had been involved in consultation and decision making, without drawing on the wealth of experience and knowledge in other parts of the local community [23].

“…strategic decisions regarding adoption and installation had been made by stakeholders operating in a top-level management capacity, whilst service providers operating at the local level had not been involved… ‘I didn’t think that the voices of experienced service providers were heard or valued’ [Local service provider].” [23], pp. 191–192)

#### CMOC 4: Where new, long-term programmes are introduced (C), having sufficient time for the initial stages creates space for the programme team to comprehensively understand the local ecosystem (M). This understanding can facilitate the building of trusting relationships between the programme team and local community members (O)

All programmes included in this review involved new teams being formed to lead the place-based work on the mental health of young people. In some cases, these teams were formed within existing community organisations or structures, while other programmes involved external academic researchers entering communities to lead the work. Given this dynamic, whereby new teams were often leading the activities, several studies underscored the importance of dedicating sufficient time during the early phases of a programme. This period is vital for cultivating trusting relationships and, in some cases, for stakeholders to overcome an initial scepticism of a new programme [17, 18, 23, 52]. Authors cautioned against work being driven by tight research timelines and underestimating the time required to engage community members and build a basis of trust and collaboration before initiating programme activities [18, 23]. This is particularly important in communities that have had negative past experiences of external programmes or research, where past disappointments or unfulfilled commitments have led to frustration or disillusionment [17, 23]. Hickey et al. [23] also highlighted the context of economic recession and funding cuts, where the new project coming in with a large funding pot led to resentment among some community professionals.

“…’there's loads of places around here that have gotten money taken off them, funds cut and stuff like that… like people were losing their jobs… that's what a lot of the jealousy was from; like why did they get 16 million?’… [Youngballymun staff member]… this process of engagement and additional implementation planning required a greater investment of time in the planning process than was originally envisaged by the programme developers.” [23], p. 191)

“First Nations’ communities are increasingly sceptical of well-intentioned visitors who witness their “lived experience” and leave behind broken promises. Communities have been promised schools, arenas, youth centres and community centres that have never manifested... There are many promises but limited action... It is therefore a requirement of involvement of ‘southerners’ in the Partnership to make a commitment for a decade.” [17], p. 248)

#### CMOC 5: Where programmes have allocated ample time (C1) and programme teams are open to learning and adapting (C2), adopting a flexible strategy in planning and programme development provides the opportunity for community voices to be heard (M). This adaptability ensures the initiative is more attuned to community needs (O1) and facilitates the integration of change within the community (O2)

Further to CMOC 4, which highlights the time required for building relationships in place-based approaches, some articles explained the ‘crucial’ requirement for programme teams to take an adaptable and evolving approach to their activities [18, 23, 52]. It was noted that, for programmes to truly reflect the needs and voices of the community, such flexibility is essential. In the case of Youngballymun in Ireland, initial strains between community service providers and the implementation teams were alleviated through demonstrating this flexibility. This approach not only enhanced engagement and satisfaction with the programme but also bolstered the community’s commitment to embedding the change [23].

“A responsive approach to implementation planning, which was characterised by flexibility and openness to change, was an important mechanism in the successful progression of exploration and installation… The process of implementation takes time, and rarely proceeds in a linear straightforward manner (Akin et al., 2017; Barnes, Matka, & Sullivan, 2003). In the case study outlined here, the allocation of sufficient time to the exploration and installation phase was important in navigating community/service provider concerns regarding innovation/change, securing buy-in and engagement for the strategy.” [23], p. 193)

“Participants described how the HeadStart programme being agile, flexible and adaptive had enabled it to work across different parts of the system and had ensured that it remained responsive and relevant to need over the six year period of its implementation… Participants referenced how the HeadStart programme had evolved over time in response to feedback from partners, local evaluation data and external events.” [52], p. 20)

#### CMOC 6: Where multiple different organisations come together for the first time (C), programme teams facilitating the development of a shared vision can help create a collective understanding of local issues and the focus of the programme (M) and may lead to a stronger foundation for partnership working (O)

When working across organisations with different priorities and agendas, it is important to create a shared vision or mission for the specific work of the place-based initiative. Several authors described facilitating workshops that brought partners together to articulate a set of collective goals for the project, along with a “sense of collective understanding of how change was assumed to occur” [12], p. 473). Others noted the importance of documenting objectives and a shared vision at the outset, while making sure this is communicated clearly and consistently [18, 23, 52]. It is important to note, however, that while authors described working towards a collective vision, the process for reaching a consensus was not explained in detail. It was also unclear at times whether this vision or mission was truly coming from within the community or rather the process was more about getting community members on board with a vision that had been developed externally.

“At the outset of the planning process, it is important to establish a shared vision which incorporates and respects the priorities and preferences of multiple voices from the local community. This vision should be shared and agreed with a broad range of key stakeholders/service providers early in the planning process and any reservations or potential barriers to implementation identified and resolved.” [23]

“HeadStart was described as a focal point for collaboration within local areas, bringing organisations and individuals together through, for example, instigating multi-agency meetings, multidisciplinary partnership boards and networking events... Participants described how good relationships were developed over time and through sustained collaborative working. HeadStart having shared goals, knowledge and purpose with other local services and organisations had facilitated this.” [52], p. 19)

### Bringing a social determinants lens

Many articles described how teams explicitly brought a social determinants lens to the work on adolescent mental health, providing community stakeholders and young people with a different way of thinking about the issue of adolescent mental health in their local area. Outcomes were mainly discussed in relation to young people, who were empowered by this knowledge, and community professionals, who shifted the way they were thinking about young people’s mental health.

#### CMOC 7: In contexts where the prevailing focus is on an individualistic model of mental health (C), introducing programmes with a deliberate emphasis on social determinants facilitates a broader understanding that encompasses environmental factors (M). This approach can help community professionals think more holistically about young people’s mental health (O)

Whether or not they used exact wording on ‘social determinants’ of health, these place-based approaches all framed youth mental health as affected by multiple factors (e.g. families, peers, geographies, media), histories (e.g. racism, colonisation, intergenerational trauma) and systemic influences (e.g. education, healthcare services, employment). Numerous studies characterised this approach to framing young people’s mental health and wellbeing as distinct from conventional service delivery or community support in the area [16, 17, 21, 29, 52]. The authors observed that this focus on social determinants and systemic influences brought about a clearer or new understanding for many stakeholders, which has either already altered or could potentially modify their future practices. Participants in HeadStart in the UK felt that the wider environment was being considered in their local areas as a result of programme activity and noted that the emphasis on contextual factors in hindering or supporting adolescent mental health had highlighted the need for a more holistic approach [52].

“Many youth and adult ally participants articulated profound shifts in their understandings of mental health, moving from an illness-oriented, biomedical framing to one that now also includes an appreciation for, and application of, the social and structural determinants.” [29], p. 11)

#### CMOC 8: In the context of a safe, trusted space (C), training young people in social determinants of mental health and providing a structured opportunity for them to share their experiences opens their eyes to wider systemic issues (M) and helps to create a collective understanding of their experience (O)

Specific initiatives, such as Agenda Gap [29], not only approached their objectives by addressing the broad underlying and structural determinants of mental health but also specifically engaged in educating young people on these topics. In the Agenda Gap project, participants received training on mental health promotion, the social and structural determinants of mental health, issues of (in)equity, and youth rights concerning policy advocacy. This educational approach aimed at empowering them to develop strategies and action plans to effect change within their communities [29]. Conducted through weekly sessions over approximately 6 months, this training and collaborative work enabled young participants to contextualise their personal experiences within the broader system and develop a more collective understanding of the factors influencing mental health.

“One participant said she came to understand, ‘how deep-rooted racism can affect mental health and how it’s not just about personal change, it’s more about community-based support.’ This new knowledge also extended their ideas about how mental health could be strengthened or promoted, including through policy change. One participant shared, ‘I have a much better understanding of how policy affects me and how it can affect youth mental health.’” [29], p. 7)

This collective understanding of their experience became the context for CMOC 9 (see Fig. 2).

#### CMOC 9: With a new understanding of social determinants (C), conversations with supportive adults about youth rights, abilities and opportunities to enact change may lead to increased confidence and communication skills (M), helping young people feel empowered to take action (O)

Building on from this new understanding around determinants of mental health, young people explained that this knowledge made them feel more empowered to engage and work to create change. Engagement in place-based programme activities that facilitate learning and discussion among peers helps to build confidence, self-esteem and leadership skills [14, 21, 29]. While most articles did not provide examples of how this youth empowerment was carried forward, some youth participants in Agenda Gap described increased motivation and participation in school projects. There were instances of students applying the principles of Agenda Gap within their educational settings and communities, with follow-up dialogues led by young people [29], p. 10). In this instance, the positive experiences with adult allies in Agenda Gap activities also built trust that young people’s contributions were valued and would be listened to [29].

“This new way of looking at mental health was further described by other participants who explained that in addition to gaining awareness of the social and structural origins of mental health and illness, their participation in Agenda Gap contributed to a shift from feeling powerless to empowered and more equipped to take action:

‘...How we could impact as youth, ‘cause a lot of youth, myself included, feel like nothing I say really matters cause it’s all adults in charge. But actually realizing that we can change things and being able to present to [decision maker in the education system] was very empowering.’” [29], p. 7)

“Some participants even went as far as to state that their sense of pride in themselves and their heritage and culture increased as a result of engaging in the project. For example… ‘I used to be so ashamed of being native and now I’m not … so yeah, it made me happy for who I am and where I come from’ [Participant 2]. Youth gained stronger connections with elders and culture and reported that this new-found awareness had a positive impact on their sense of identity.” [21], p. 27)

### Educating and empowering community stakeholders

Some of the articles explained how participation in this type of place-based work was educational and empowering for community stakeholders (namely young people and community professionals working with young people). However, there was limited detail on how this empowerment takes place and any ensuing outcomes.

#### CMOC 10: Where adults are committed to centring youth voice (C), offering them training in participation models and facilitation skills improves confidence and clarity of roles (M), enabling them to employ strategies to create safe and empowering spaces for young people (O)

All included articles described a context whereby those initiating or leading the place-based approach were committed to integrating the views of young people into their work. This belief in creating space for and incorporating the opinions and experiences of local young people underpinned each programme. However, authors noted that adult facilitators require a specific set of knowledge and skills to engage young people in a meaningful way, and locally relevant training is required [5, 57, 58]. Belief in the value of youth voice is not enough to do this work successfully and it should not be assumed that local professionals (e.g. social workers, teachers, youth workers, health service staff) will already have the necessary skills [5].

“The majority of adult allies believed that they simply needed to “get out of the way” to allow young people to lead... While it was evident that this belief was intended to highlight the value adults placed on youth voice, it also undermined the adult role in the initiative. In an effort to allow young people to lead, adult allies were not providing the leadership and support needed for young people be successful.” [5], p. 47)

“Provide training on youth/adult partnerships to both youth and adults: Regional staff determined early on that effectively implementing productive and mutually beneficial youth/adult partnerships at all levels of the Coalition required training on this topic.” [58], p. 179)

This safe and empowering space was a key context for CMOC 10 (see Fig. 2).

#### CMOC 11: Where system leaders traditionally do not involve youth in strategic planning or decision-making processes (C), providing opportunities to see the process and outputs from activities with young people means they may see the value of youth voice (M) and shift their practice to include young people in mental health strategy design (O)

Several articles sought to create structural change through illustrating to system leaders the importance and benefits of including local youth voices in mental health planning and strategy [17, 29, 52]. While some authors portrayed this effort as a significant challenge to system leaders [25], a healthcare decision-maker involved in Agenda Gap reported the experience had motivated them to advocate for the meaningful engagement of young people in other areas of their work [29]. Having the opportunity to be involved in youth voice activities and participatory research may encourage more community professionals and policymakers to prioritise this in future work and mental health strategy design. Some areas that participated in HeadStart saw the programme as leading and demonstrating best practice in participation and co-production work, with participants linking this to a new focus on young people’s rights and voice in their local councils [52].

“It really turned up the volume on my intention [to be] curious about the youth’s experience and being curious about their strengths and really advocating strongly in meetings… [Engaging with Agenda Gap] was just such a good reminder of all the strengths and wisdom that youth bring… We’ve been talking a lot about policy level and program development and starting new teams in my area of practice. And this way of thinking I’d say, has been embedded in all of those. So, in some ways that’s a tangible outcome or difference.” [29], p. 10)

“Young people expressed their views simply, directly, and without the scepticism that can often infect conversations about change. In their contributions to planning meetings, young people brought disarming freshness that grounded the discussion. They reminded those present that the reason for the gathering was not primarily to benefit services, but to better engage with young people. Involving young people in plan design challenged service providers who had minimal previous experience of engaging with young people in this way” [25], p. 427)

## Discussion

The aim of this review was to develop our understanding of the mechanisms through which place-based approaches work to improve the mental health and wellbeing of young people. Despite growing interest in these strategies for addressing adolescent mental health concerns in recent years, there is limited understanding of how these programmes work, for whom and under what circumstances. As a first step in bridging this knowledge gap, we identified 11 CMO configurations across three primary domains in 12 place-based approaches: building relationships and trust; bringing a social determinants lens; and educating and empowering community stakeholders. Our CMOCs offer unique contributions to understanding the mechanisms through which place-based approaches are operating. The findings illustrate important considerations for a range of stakeholders, from young people to system leaders, with a particular emphasis on the programme teams initiating and leading the work.

Although we did not set out to examine the interactions between CMOCs in this review, we observed notable ‘ripple effects’ [22], whereby outcomes resulting from programme activities become the new context for other activities or mechanisms. Ripple effects were especially apparent in CMOCs related to adults facilitating youth voice and participation and in their programme activities (see Fig. 2). CMOC 10 highlights the significance of training adults in participatory models and facilitation techniques to establish safe and empowering environments for youth. These safe and empowering spaces, in turn, provide the context for CMOC 8, where young people can learn and talk about the social determinants of mental health and create a collective understanding of their experiences. Finally, CMOC 9 shows how this new understanding of social determinants is combined with discussions about opportunities to enact change, which then empowers and motivates young people to initiate change. The time and resources required to achieve outcomes such as a safe and empowering space for youth involvement cannot be underestimated, and yet it is a necessary step before further problem-solving and action can take place. Viewing these configurations as a series of ripple effects can be helpful to understand how programme activities progressively build upon each other and to demonstrate the time required to move through different stages of a project to reach certain outcomes [27]. The example here has implications for the design and structure of place-based approaches,programme developers must carefully consider their schedule of activities and the resources required for work to take place over time [11].

The articles reviewed and the resultant CMOCs primarily address the intermediate outcomes of these place-based programmes, such as engaging the right stakeholders and creating opportunities for youth voices to be heard. However, there remains a question of what happens next; what is the pathway to wider systemic change? The strategies and activities detailed in the included articles, which were implemented through place-based approaches, generally focus on smaller-scale interventions, such as establishing youth centres or cafes, conducting local radio campaigns to raise awareness, or organising stigma reduction workshops in schools. There appears to be a significant challenge in translating the types of activities described (e.g. forming coalitions or partnerships, integrating youth perspectives) into tangible or measurable structural or systemic improvements in mental health services. This challenge is also recognised in wider literature on place-based approaches, where authors highlight the pull towards individual solutions and actions [2, 7, 30]. While many articles included in this review discussed hopes and intentions regarding longer-term and systemic goals, none of them had been able to follow-up and explore these outcomes, leaving our understanding of the pathway to achieve systemic change requiring further attention.

Similarly, there was very little detail in these articles regarding ownership and sustainability of these place-based approaches; while authors described the involvement of community members and the desire for long-term implementation, there was either limited or no discussion regarding future plans or processes that would support this. A recent review on the sustainability of community mental health assets found significant emphasis on the availability and diversity of funding, with a lack of funding limiting organisational activities and reducing programme capacity [40]. Economic uncertainty and corresponding challenges for community organisations around long-term funding also emerged as key factors affecting sustainability, and yet plans for securing sustained funding were not addressed by authors of the included studies. It may be that programme leaders hoped the participatory nature of the work would lead to capacity building and the potential for communities to sustain project goals, as has been noted in other research on participatory approaches [27, 28]. In their realist evaluation of community-based participatory research in the field of healthcare, Jagosh et al. [27] found that the trust and capacity building that takes place in successful partnership approaches can make substantial contributions to sustainability and systems transformation. However, in the articles reviewed here, the foundations for sustainability were not made explicit, though authors all underscored the need for a significant time investment and sustained input to create change and shift practices in a place. In wider literature on place-based work, authors have highlighted the need for work to advance at the speed of trust in the community and have suggested that it can take over 10 years to see population-level outcomes [11]. Further long-term research is required to develop our understanding of the underlying mechanisms that lead to systems change and the sustainability of these types of place-based initiatives to improve adolescent mental health.

Programmes in other areas of health have reported similar challenges regarding sustainability and success in developing solutions that lead to systemic change. Recently, the large-scale CO-CREATE project employed a youth-led participatory action research approach to generate policy ideas towards the reduction of adolescent overweight and obesity across Europe [31]. Despite taking a complex systems perspective and using a range of methods to incorporate systems thinking into the work, 86% of the policy ideas generated by a co-design process with young people were categorised as operational rather than systems level [7]. Examples of these operational suggestions include cooking classes in schools, free fruit in school canteens, smaller portion sizes and adverts that promote positive body image [7]. The authors call for more use of tools such as the Intervention Level Framework [30, 37] to help operationalise systems thinking, and have also proposed a protocol for embedding systems thinking into research [32]. These tools may help those delivering and evaluating place-based approaches to structure thinking about impact at the system level and maintain a focus on some of the more challenging levels of the system at which to intervene.

Employing theories like complexity theory to guide place-based approaches might also be useful given the scale and complex nature of these approaches [53]. Many of the studies included in this review draw on systems theories to describe relational or mindset shifts for individuals within and across systems. In their protocol, Freebairn et al. [18] use a participatory systems modelling approach as a framework for their activities, noting how systems approaches recognise that mental health issues occur within wider systems that are dynamic and need to be better understood in order to make impactful change. Similarly, the HeadStart programme set out with a systems change approach, aiming to alter underlying structures and work as a catalyst to reshape the existing mental health system in the UK [52]. Several articles also used ecological theories such as Bronfenbrenner’s [3] framework in their place-based approaches to acknowledge that young people are embedded in and affected by multiple and interconnecting systems [12, 25, 34]. However, complexity theory builds on these ideas to view systems as non-linear and unpredictable [53]. Complexity theory goes further than systems theory, operating on the principle that the whole is different from the sum of its parts and including the ideas of self-organisation and emergence,interactions between the system’s constituent parts lead to new emergent properties or behaviours which, in turn, feed back into the behaviour of individuals [13, 38]. Concepts such as emergence are particularly important for both delivering and evaluating place-based approaches, as they allow the exploration of unpredictable outcomes that may influence system dynamics.

### Limitations

This review is one of the first to bring together literature on place-based approaches to address adolescent mental health, highlighting theories which can be employed by programme developers and researchers in the future. However, while this review was systematic, the nature of a rapid realist review means that the search was not exhaustive. Additionally, despite seeking and including grey literature in this review, it was difficult to follow up on most of these place-based initiatives included after publication; many websites referenced within articles no longer exist and, in many cases, it was not possible to access project reports. This limits the possibility of exploring important concepts such as ownership and sustainability, and the lack of longitudinal studies across the review makes it difficult to evaluate systemic impact. Many of the included articles were also from Western countries and contexts, with well-established health service structures and policies on mental health. Though the mechanisms are likely similar across countries and contexts, this may limit the transferability of some of the CMOCs to other settings.

## Conclusions

This review provides valuable insights into the mechanisms underlying place-based approaches seeking to improve adolescent mental health and wellbeing. The findings have usefully informed and directed the developmental evaluation of the Kailo programme in the UK [24], providing key mechanisms of focus and routes of further enquiry. However, there remain several questions around how such programmes sustain their processes and activities over time, and how to translate operational or intermediate outcomes successfully into systemic change. Given the global demand for effective support for adolescent mental health and wellbeing, further thought and attention is required to explore ways to create meaningful and sustainable change.

## Supplementary Information

Additional file 1: RAMESES Reporting Guidelines.

Additional file 2: Search terms.

## Data Availability

All data generated or analysed during this study are included in this published article and its supplementary information files.

## Footnotes

1 Given the nature of this work, whereby we are synthesising information across multiple studies and contexts, we have used the term ‘Indigenous communities’ here to refer to several different First Nations, Aboriginal and Torres Strait Islander peoples across Canada and Australia. We recognise that using an umbrella term is never ideal and acknowledge the diversity of culture, language, beliefs and practices across these communities.
