# Supplementary material for: Place-based approaches to improve the mental health and wellbeing of children and young people: a rapid realist review
Source: Syst Rev. 2025 May 19;14:115. doi: 10.1186/s13643-025-02838-8 (PMC12090407; doi:10.1186/s13643-025-02838-8)
Supplement: Supplementary file 2 — Additional file 2: Search terms. [file 13643_2025_2838_MOESM2_ESM.docx]

**Additional File 2 – Search terms**

**Database: PsycINFO (via Ovid)**

| **#** | **Search query** |
| --- | --- |
| 1 | (young* adj2 (man* or men* or woman* or women* or person* or people* or population* or adult* or individual*)).ti. |
| 2 | (youth* or youngster* or adolescen* or teen*).ti. |
| 3 | ((emerging or early) adj2 adult*).ti. |
| 4 | 1 or 2 or 3 |
| 5 | ((place or area* or communit* or location* or partnership* or neighbourhood* or neighborhood*) adj2 (level or initiative* or approach* or development* or base* or intervention)).tw. |
| 6 | ((communit* or neighbourhood* or neighborhood*) adj2 partnership*).tw. (2828) |
| 7 | ((collaborativ* or participat* or action or codesign* or co design* or cocreat* or co creat*) adj2 research).tw. |
| 8 | exp action research/ |
| 9 | 5 or 6 or 7 or 8 |
| 10 | 4 and 9 |
| 11 | (mental* or disorder* or wellbeing or anxi* or emotion* or psych* or depress* or panic* or phobi* or trauma* or problem* or issue* or difficult* or alcohol* or misuse* or abuse* or substance* or stress* or withdraw* or resilien* or wellbeing or "well being").tw. |
| 12 | *mental health/ or exp youth mental health/ |
| 13 | 11 or 12 |
| 14 | 10 and 13 |
| 15 | (change* or system* or model*).tw. |
| 16 | 14 and 15 |
| 17 | (young* adj2 (man* or men* or woman* or women* or person* or people* or population* or adult* or individual*)).tw. |
| 18 | (youth* or youngster* or adolescen* or teen*).tw. |
| 19 | ((emerging or early) adj2 adult*).tw. |
| 20 | 17 or 18 or 19 |
| 21 | ((place or area* or communit* or location* or partnership* or neighbourhood* or neighborhood*) adj2 (level or initiative* or approach* or development* or base* or intervention)).ti. |
| 22 | ((communit* or neighbourhood* or neighborhood*) adj2 partnership*).ti. |
| 23 | ((collaborativ* or participat* or action or codesign* or co design* or cocreat* or co creat*) adj2 research).ti. |
| 24 | 21 or 22 or 23 |
| 25 | 20 and 24 |
| 26 | 13 and 25 |
| 27 | 15 and 26 |
| 28 | 16 or 27 |
| 29 | 4 and 9 and 24 |
| 30 | 28 or 29 |
| 31 | limit 30 to yr="2000 -Current" |

**Database: Social Science Citation Index, Social Sciences and Humanities Conference Proceedings, and Emerging Sources Citation Index (via Web of Science)**

| # | Search Query |
| --- | --- |
| 1 | TI=(young* NEAR/2 (man* or men* or woman* or women* or person* or people* or population* or adult* or individual*)) Editions: WOS.SSCI,WOS.ISSHP,WOS.ESCI |
| 2 | TI=(youth* or youngster* or adolescen* or teen*) Editions: WOS.SSCI,WOS.ISSHP,WOS.ESCI |
| 3 | TI=((emerging or early) NEAR/2 (adult*)) Editions: WOS.SSCI,WOS.ISSHP,WOS.ESCI |
| 4 | #3 OR #2 OR #1 Editions: WOS.SSCI,WOS.ISSHP,WOS.ESCI |
| 5 | TS=((place or area* or communit* or location* or partnership* or neighbourhood* or neighborhood*) NEAR/2 (level or initiative* or approach* or development* or base* or intervention)) Editions: WOS.SSCI,WOS.ISSHP,WOS.ESCI |
| 6 | TS=((communit* or neighbourhood* or neighborhood*) NEAR/2 (partnership*)) Editions: WOS.SSCI,WOS.ISSHP,WOS.ESCI |
| 7 | TS=((collaborativ* or participat* or action or codesign* or "co design" or cocreat* or "co create") NEAR/2 (research)) Editions: WOS.SSCI,WOS.ISSHP,WOS.ESCI |
| 8 | #7 OR #6 OR #5 Editions: WOS.SSCI,WOS.ISSHP,WOS.ESCI |
| 9 | #8 AND #4 Editions: WOS.SSCI,WOS.ISSHP,WOS.ESCI |
| 10 | TS=(mental* or disorder* or wellbeing or anxi* or emotion* or psych* or depress* or panic* or phobi* or trauma* or problem* or issue* or difficult* or alcohol* or misuse* or abuse* or substance* or stress* or withdraw* or resilien* or wellbeing or "well being") Editions: WOS.SSCI,WOS.ISSHP,WOS.ESCI |
| 11 | #9 AND #10 Editions: WOS.SSCI,WOS.ISSHP,WOS.ESCI |
| 12 | TS=(change* or system* or model*) Editions: WOS.SSCI,WOS.ISSHP,WOS.ESCI |
| 13 | #11 AND #12 Editions: WOS.SSCI,WOS.ISSHP,WOS.ESCI |
| 14 | TI=((place or area* or communit* or location* or partnership* or neighbourhood* or neighborhood*) NEAR/2 (level or initiative* or approach* or development* or base* or intervention)) Editions: WOS.SSCI,WOS.ISSHP,WOS.ESCI |
| 15 | TI=((communit* or neighbourhood* or neighborhood*) NEAR/2 (partnership*)) Editions: WOS.SSCI,WOS.ISSHP,WOS.ESCI |
| 16 | TI=((collaborativ* or participat* or action or codesign* or "co design" or cocreat* or "co create") NEAR/2 (research)) Editions: WOS.SSCI,WOS.ISSHP,WOS.ESCI |
| 17 | #14 OR #15 OR #16 Editions: WOS.SSCI,WOS.ISSHP,WOS.ESCI |
| 18 | TS=(young* NEAR/2 (man* or men* or woman* or women* or person* or people* or population* or adult* or individual*)) Editions: WOS.SSCI,WOS.ISSHP,WOS.ESCI |
| 19 | TS=(youth* or youngster* or adolescen* or teen*) Editions: WOS.SSCI,WOS.ISSHP,WOS.ESCI |
| 20 | TS=((emerging or early) NEAR/2 (adult*)) Editions: WOS.SSCI,WOS.ISSHP,WOS.ESCI |
| 21 | #18 OR #19 OR #20 Editions: WOS.SSCI,WOS.ISSHP,WOS.ESCI |
| 22 | #21 AND #17 Editions: WOS.SSCI,WOS.ISSHP,WOS.ESCI |
| 23 | #22 AND #10 Editions: WOS.SSCI,WOS.ISSHP,WOS.ESCI |
| 24 | #23 AND #12 Editions: WOS.SSCI,WOS.ISSHP,WOS.ESCI |
| 25 | #24 OR #13 Editions: WOS.SSCI,WOS.ISSHP,WOS.ESCI |
| 26 | #4 AND #10 AND #17 Editions: WOS.SSCI,WOS.ISSHP,WOS.ESCI |
| 27 | #25 OR #26 Editions: WOS.SSCI,WOS.ISSHP,WOS.ESCI |
| 28 | #25 OR #26 and 2023 or 2022 or 2021 or 2020 or 2019 or 2018 or 2017 or 2016 or 2015 or 2014 or 2013 or 2012 or 2011 or 2010 or 2009 or 2008 or 2007 or 2006 or 2005 or 2004 or 2003 or 2002 or 2001 or 2000  (Publication Years) Editions: WOS.SSCI,WOS.ISSHP,WOS.ESCI |
